# Persistent syphilitic ocular manifestations despite treatment: a case series

**DOI:** 10.1186/s12348-024-00435-9

**Published:** 2024-10-18

**Authors:** Sairi Zhang, Kaersti L. Rickels, Vignesh Krishnan, Sami H. Uwaydat

**Affiliations:** 1 College of Medicine, University of Arkansas for Medical Sciences, Little Rock, AR USA; 2 Department of Ophthalmology, Jones Eye Institute, University of Arkansas for Medical Sciences, Little Rock, AR USA

**Keywords:** Syphilis, Syphilitic outer retinopathy, Posterior placoid chorioretinopathy, Neurosyphilis

## Abstract

**Background:**

Penicillin has remained the most effective treatment for syphilis for several decades. Syphilitic retinal manifestations may persist following treatment and cause visual problems. In this case series, we describe three syphilis patients with persistent posterior segment manifestations due to chronic inflammation, incomplete treatment, and reinfection.

**Case series:**

Recommended initial treatment for all patients was 14 days of intravenous penicillin. Oral prednisone was added 48 h after initiation of penicillin therapy. Case 1: A 48-year-old female presented with gradual vision loss for two months. Fundus imaging revealed syphilitic outer retinopathy (SOR), papillitis, and acute syphilitic posterior placoid chorioretinopathy (ASPPC). After treatment, she had persistent cystoid macular edema (CME) and was treated with intravitreal triamcinolone injections and ketorolac drops. Case 2. A 24-year-old male presented with sudden vision loss for two days. On imaging, he had ASPPC, papillitis, and SOR. IV penicillin treatment was given for 10 days only. He had persistent SOR and was retreated with doxycycline and prednisone. Case 3: A 52-year-old male presented with eye pain and visual loss for one week. There was evidence of ASPPC and papillitis on imaging. One month after treatment, he had persistent papillitis and was restarted on oral prednisone. One year later, he was found to have recurrent ASPPC and was confirmed to be reinfected with syphilis, for which he was retreated.

**Conclusion:**

When treating persistent syphilitic ocular manifestations, we recommend checking that the penicillin treatment was complete and the RPR titers are declining. If both hold true, then the affected eye should be treated with anti-inflammatory therapy. Other factors that contribute to poor visual prognosis include treatment delay, poor initial visual acuity, macular edema, and HIV coinfection.

## Background

Syphilis is a sexually transmitted infection caused by *Treponema pallidum* that can present with a wide variety of ocular manifestations such as uveitis, keratitis, retinal vasculitis, and optic neuropathy [1]. Ocular manifestations can arise at any stage of syphilis, and clinical presentation is highly variable and can mimic that of other ocular diseases. Posterior segment findings include syphilitic outer retinopathy (SOR), acute syphilitic posterior placoid chorioretinopathy (ASPPC), retinitis, chorioretinitis, and papillitis [2, 3]. Diagnosis is clinical with both treponemal and non-treponemal serological testing.

Penicillin has remained the gold standard of syphilis treatment since its first use in 1943. Ocular syphilis is treated identically to neurosyphilis with 10 to 14 days of intravenous (IV) penicillin G, 4 million units every 4 h [4], and often followed by 1–2 weeks of oral penicillin outpatient. *T. pallidum* can reside in sequestered sites such as ocular structures, so the use of parenteral rather than intramuscular penicillin G is critical [5]. Ocular syphilis can be treated effectively with penicillin, although delayed diagnosis and management may lead to poor visual outcomes [6].

To date, no cases of syphilitic resistance to penicillin have been reported, and the emergence of penicillin resistance is unlikely due to the mutational changes required [5]. However, there have been reports of persistent *T. pallidum* in syphilis patients following treatment, particularly in the aqueous humor [7–9]. Cases of neurosyphilis persisting despite penicillin therapy have also been reported [10, 11]. In addition, treatment failure with inadequate serologic response has been reported for primary and secondary syphilis [12]. Nevertheless, it is rare for complete treatment with penicillin to be ineffective, although chronic uveitis and persistent visual problems are still possible after treatment [13].

In this case series, we present three patients whose syphilitic posterior segment manifestations persisted despite treatment with parenteral penicillin, either due to chronic inflammation, incomplete treatment, or reinfection.

### Case 1

**Fig. 1:**
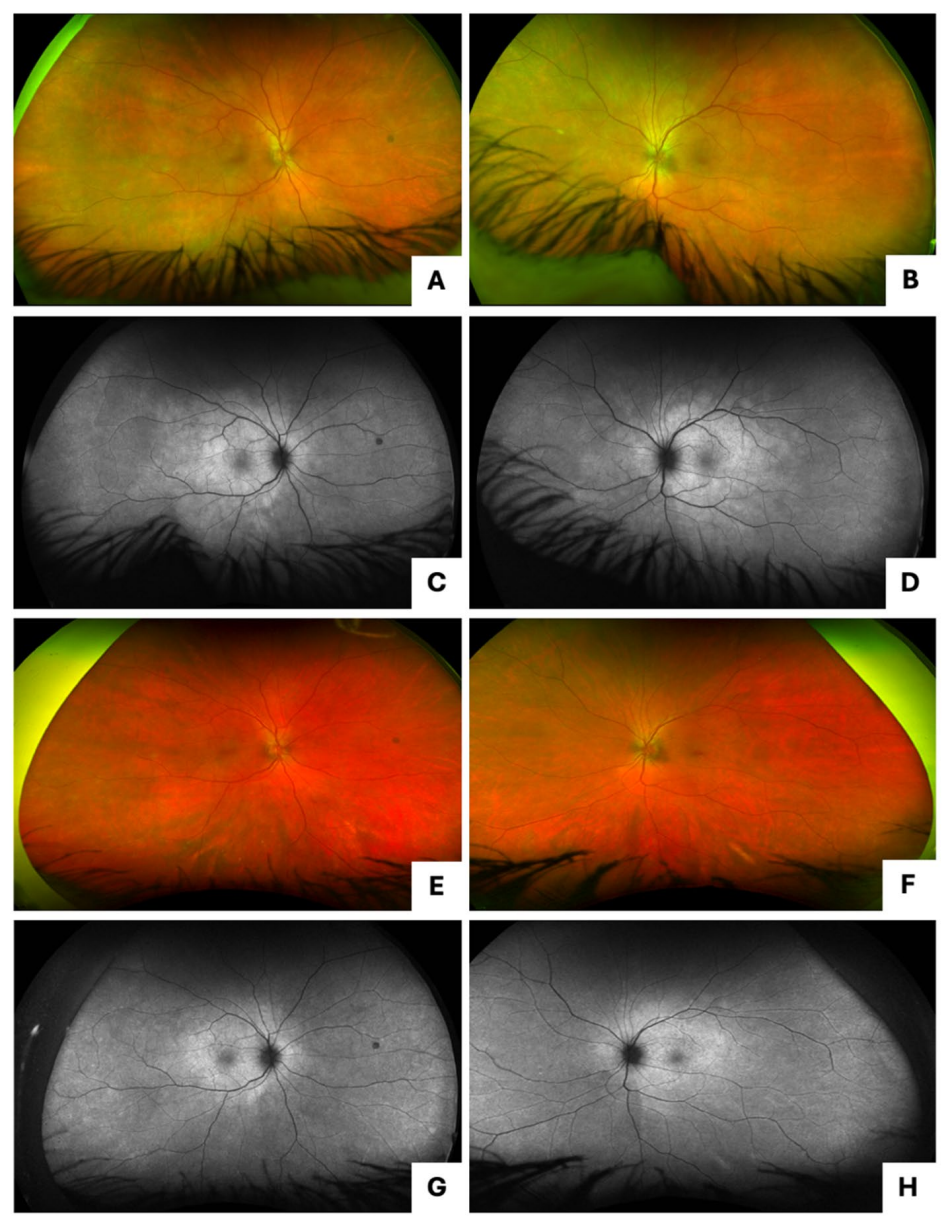
Case 1 presentation imaging (**A**, **C**) pseudocolor and fundus autofluorescence imaging of the right eye with SOR and papillitis; (**B**, **D**) pseudocolor and fundus autofluorescence imaging of the left eye with SOR, papillitis, and ASPPC; Case 1 five months after presentation (**E**, **G**) pseudocolor and fundus autofluorescence imaging of the right eye with improved SOR; (**F**, **H**) pseudocolor and fundus autofluorescence imaging of the left eye with improved SOR

**Fig. 2:**
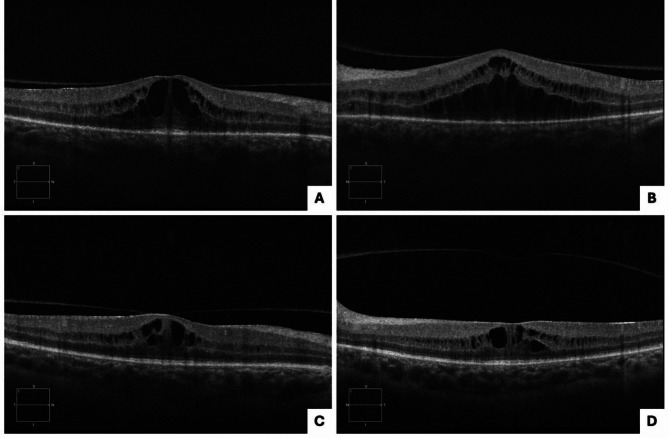
Case 1 presentation optical coherence tomography (OCT): cystoid macular edema (CME), retinal pigment epithelium (RPE) nodules, and disruption of the ellipsoid zone (EZ) and external limiting membrane (ELM) layers noted OU (**A**: right eye), (**B**: left eye); Case 1 five months after presentation on OCT: persistent CME noted OU (**C**: right eye), (**D**: left eye)

A 48-year-old female with a history of IV methamphetamine abuse presented with gradual vision loss for two months. Initial visual acuity (VA) was [20/40] in the right eye (OD) and [20/40] in the left eye (OS). Fundus autofluorescence (FAF) and pseudocolor imaging revealed SOR and papillitis in both eyes (OU) and ASPPC OS (Fig. 1A-D), which was confirmed with indirect ophthalmoscopy. On optical coherence tomography (OCT), cystoid macular edema (CME), retinal pigment epithelium (RPE) nodules, and disruption of the ellipsoid zone (EZ) and external limiting membrane (ELM) layers were noted OU (Fig. 2A-B). The patient tested positive for syphilis with both positive *T. pallidum* IgG antibody and rapid plasma reagin (RPR) at 1:16. She was admitted for neurosyphilis workup and was treated with IV penicillin for a total of 14 days. Oral prednisone 40 mg daily was added 48 h after initiation of penicillin therapy and continued for two weeks. The patient reported improvement in vision following the initiation of treatment.

One month later, she had persistent CME OU on examination. She received an intravitreal triamcinolone injection OS, after which there was some improvement. However, the patient still had persistent SOR on FAF and CME on OCT. Five months after the initial diagnosis, RPR was still positive at 1:8. Due to concern for inadequate initial treatment, penicillin therapy was restarted for three weeks. The patient’s SOR improved (Fig. 1E-H), but she had persistent CME OU (Fig. 2C-D) and was started on ketorolac drops OU. At her next visit, her CME was resolved OS but remained persistent OD, so she received another intravitreal triamcinolone injection OD. The patient’s VA consistently remained between [20/30] and [20/40] OU throughout her follow-up. She was eventually lost to follow-up.

### Case 2

**Fig. 3:**
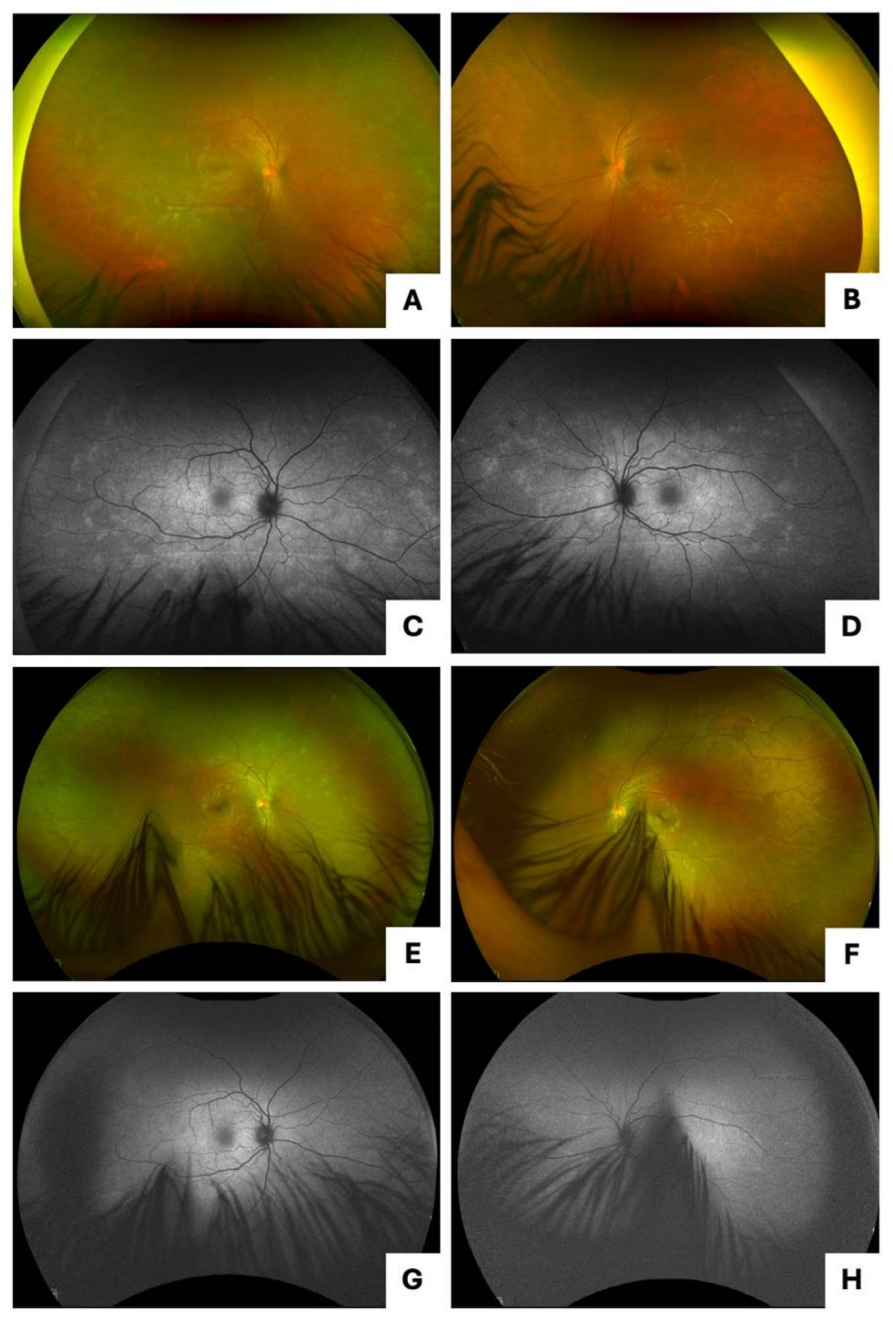
Case 2 presentation imaging (**A**, **C**) pseudocolor and fundus autofluorescence imaging of the right eye with ASPPC and papillitis; (**B**, **D**) pseudocolor and fundus autofluorescence imaging of the left eye with ASPPC, SOR, and papillitis; Case 2 five months after presentation (**E**, **G**) pseudocolor and fundus autofluorescence imaging of the right eye with persistent SOR and areas of perivasculitis; (**F**, **H**) pseudocolor and fundus autofluorescence imaging of the left eye with resolved retinitis

**Fig. 4:**
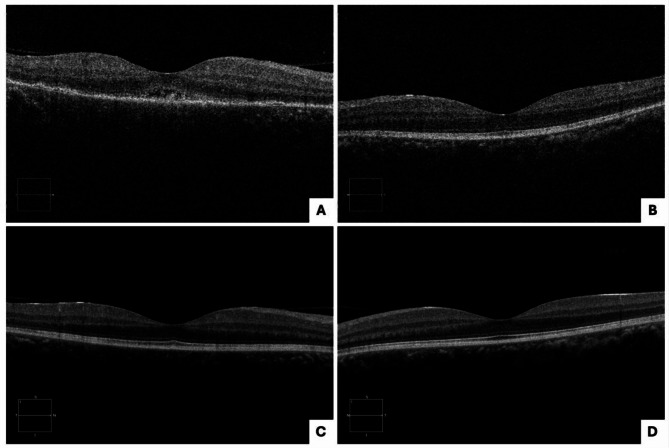
Case 2 presentation optical coherence tomography (OCT): retinal pigment epithelium (RPE) nodules, and disruption of the ellipsoid zone (EZ) and external limiting membrane (ELM) layers noted OU (**A**: right eye), (**B**: left eye); Case 2 five months after presentation on OCT: normalized macula findings OU (**C**: right eye), (**D**: left eye)

A 24-year-old previously healthy male presented to the clinic with sudden vision loss OD for two days, in addition to floaters and flashes for several months. VA was counting fingers (CF) at 5 feet OD and [20/25] OS. FAF and pseudocolor imaging revealed ASPPC and papillitis OU and SOR OS (Fig. 3A-D). On OCT, RPE nodules and disruption of the EZ and ELM layers were noted OD (Fig. 4A-B). The patient tested positive for syphilis with positive *T. pallidum* IgG antibody and RPR at 1:64. He was admitted and completed a 10-day course of IV penicillin and oral prednisone with significant improvement.

Five months later, the patient’s VA was [20/20] OD and [20/25] OS, and he had persistent SOR OD and resolved retinitis OS (Fig. 3E-H). OCT macula findings normalized at the five-month visit (Fig. 4C-D). He was additionally treated with oral doxycycline 100 mg twice daily for one month. Oral prednisone 40 mg daily was restarted for two weeks. Two weeks later, no signs of active retinitis or uveitis were noted, so prednisone was tapered. One month later, FAF showed resolution of the SOR. Repeat RPR was non-reactive. After one year, the patient’s VA was [20/20] OU, and fundus findings and imaging were all normal.

### Case 3

A 52-year-old male with a history of human immunodeficiency virus (HIV) untreated for over a year, tobacco use, and methamphetamine abuse presented with eye pain and visual loss OD for over a week. VA was hand motion (HM) OD and [20/25] OS. *T. pallidum* IgG antibody was positive and RPR was reactive at > 1:128. Lumbar puncture was deferred, and MRI of the brain and orbits was normal. The patient was started on a 14-day course of penicillin, in addition to oral prednisone, prednisolone drops, and atropine drops.

**Fig. 5:**
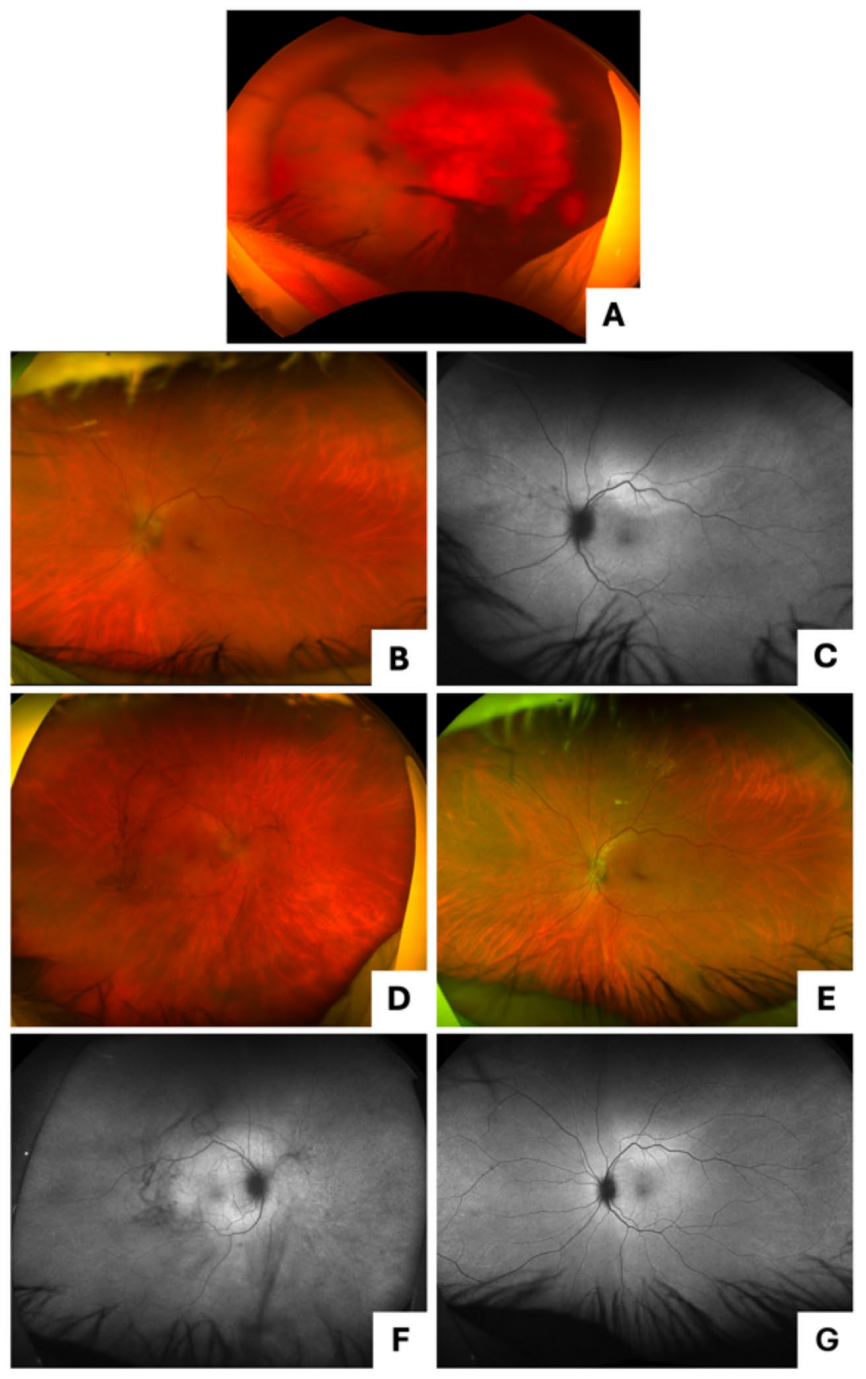
Case 3 presentation imaging (**A**) pseudocolor imaging of the right eye with dense vitritis obstructing view of the retina; (**B**, **C**) pseudocolor and fundus autofluorescence imaging of the left eye with ASPPC and papillitis; Case 3 one month after presentation (**D**, **F**) pseudocolor and fundus autofluorescence imaging of the right eye with persistent papillitis and abnormal vessels temporally; (**E**, **G**) pseudocolor and fundus autofluorescence imaging of the left eye

At clinic follow-up one week after presentation, the patient’s VA was HM OD and [20/40] OS. There was no clear view of the retina OD due to obstruction by dense vitritis (Fig. 5A). FAF and pseudocolor imaging showed evidence of ASPPC and papillitis OS (Fig. 5B-C), which were confirmed on indirect biomicroscopy. At the one-month visit after completing treatment, the patient’s VA improved to [20/30] OD and [20/25] OS, but he had persistent papillitis OD (Fig. 5D-F) and was subsequently restarted on oral prednisone. RPR titer was 1:16. One year later, his VA worsened again to HM OD and [20/30] OS. On imaging, he had ASPPC OU. RPR had increased to 1:128, suggesting reinfection with syphilis. Inpatient treatment with 14 days of IV penicillin and oral prednisone was started. After treatment, the patient’s VA improved to [20/60] OD and [20/25] OS. There was mild CME noted OD. The patient received a triamcinolone injection OD to mitigate the continued inflammation; however, the patient was lost to follow-up before convalescence was confirmed.

Table 1 summarizes the relevant findings for each patient, including past medical history, presenting symptoms, initial exam findings, treatment course, and patient outcome. Table 2 summarizes the progression of RPR titers following initiation of treatment for each patient in our study.

**Table 1:** Summary of relevant findings

Case	History	Symptoms	Initial Exam	Treatment	Outcome
**1**	IV meth use	Gradual vision loss OU for 2 months	VA 20/40 OD, 20/40 OS Optic disc edema, dilated venules, yellow spots in the periphery	IV penicillin and PO prednisone for 14 days Triamcinolone injection OS Penicillin for 3 weeks Ketorolac drops OU Triamcinolone injection OD	Persistent chorioretinitis and macular edema VA 20/40 OD, 20/30 OS at last visit Lost to follow-up
**2**	Previously healthy	Sudden vision loss OD for 2 days; floaters for 3 months, flashes for 1–2 months	VA CF at 5’ OD, 20/25 OS Partial peripheral CVF defects OD Optic disc edema OU	IV penicillin and PO prednisone for 10 days PO doxycycline for 1 month PO prednisone for 2 weeks	Persistent retinitis VA 20/20 OD, 20/20 OS at last visit Complete resolution after retreatment
**3**	HIV, tobacco use, meth use	Eye pain and visual loss OD for over a week; floaters, headache	VA HM OD, 20/40 OS Fixed pupil OD 4 + cell/fare and fibrin in AC with posterior synechiae OD Optic disc edema with superior hemorrhage OS	IV penicillin and PO prednisone for 14 days PO prednisone Penicillin and prednisone Triamcinolone injection OD	Syphilis recurrence VA 20/50 OD, 20/25 OS at last visit Lost to follow-up

IV, intravenous; HIV, human immunodeficiency virus; OD, right eye; OS, left eye; OU, both eyes; VA, visual acuity; CF, counting fingers; CVF, confrontational visual fields; HM, hand motion; AC, anterior chamber; PO, oral

**Table 2:** Summary of RPR titer values from diagnosis (Month 0) until months after treatment

Case 1	Month 0–1:16 Month 3–1:16 Month 4–1:8 Month 7–1:4
Case 2	Month 0–1:64 Month 4–1:64 Month 5–1:16 Month 12–1:16
Case 3	Month 0 – >1:128 Month 6–1:16 Month 12–1:128* Month 20–1:32 Month 27–1:8

*Time of reinfection with syphilis

## Discussion

This case series presents three patients with persistent syphilitic posterior segment manifestations despite appropriate treatment with intravenous penicillin and oral steroids per standard of care guidelines [12]. Each case represents an example of persistent retinal manifestations of syphilis due to a different reason. Case 1 demonstrates chronic inflammation, as evidenced by the patient’s persistent CME. Case 2 demonstrates incomplete treatment since the patient received a shorter course of penicillin and required an additional course of antibiotics and steroids to achieve convalescence. Case 3 demonstrates reinfection, which was supported by the patient’s increase in RPR titers.

From our case series, we propose three important points to consider when evaluating patients with persistent retinal manifestations of syphilis. First, confirm that the initial treatment with penicillin was complete. Second, monitor the RPR titers and ensure that they are dropping. Finally, if both of the previous conditions are met, proceed to treat the affected eye for chronic inflammation. Treatment includes corticosteroid therapy with eye drops or intravitreal injections, the latter of which has been reported to successfully treat CME in ocular syphilis, though the risk of reactivating infection with corticosteroids remains [14].

There are several other factors to consider when treating ocular syphilis patients. Studies have shown a worse visual prognosis with a delay of more than 28 days between the onset of ocular symptoms and the initiation of treatment [15, 16]. Of our three patients, two presented after at least two months of ocular symptoms. The exception in our cohort was the patient in Case 3 who presented with ocular symptoms for only a week. It is worth noting that this patient had HIV with delayed antiretroviral therapy, as discussed later.

Other factors reported to be associated with poor visual prognosis include the presence of macular edema and poor initial visual acuity [16, 17]. According to Moradi et al., visual loss and cystoid macular edema are common even after treatment with intravenous penicillin among HIV-negative patients [18]. All three patients in our study had poor initial visual acuity, the worst of which was HM. The patient in Case 1 had CME following treatment that persisted until her last follow-up, despite additional antibiotic and anti-inflammatory treatment.

Case was notable for the patient’s HIV-positive status. His untreated HIV disease could have played a role in his reinfection, as HIV coinfection has been associated with an increased risk of ocular syphilis relapse [19]. Recurrences can present with vitritis and CME, though some patients may not have any ocular symptoms [16, 19]. Therefore, regular ophthalmological follow-up after treatment and confirmation of treatment success with serological testing is crucial, as detecting reinfection and beginning retreatment can improve visual prognosis [16].

Previous literature emphasizes the importance of monitoring treatment response with RPR titers at 6 and 12 months after treatment, and a four-fold drop in titers is considered an adequate response [4]. All three of our patients achieved a four-fold drop in titers by 12 months, supporting adequate treatment responses (Table 2). Lumbar puncture and cerebrospinal fluid examination are also recommended [13, 16]; however, it was not always performed in our patients.

While RPR titer levels can demonstrate an adequate response, they may not always accurately reflect disease progression or resolution. For example, the patients in Cases 1 and 2 had persistent SOR despite their declining titers. In their systematic review, Seña and colleagues found that 12.1% of syphilis patients treated with recommended therapy did not achieve the expected four-fold decrease in titer within 12 months after treatment [20]. This is referred to as serological non-response, or the serofast state, in which there is a less than four-fold decline in nontreponemal titers or persistently low positive titers [20]. The non-response could indicate treatment failure, reinfection, or a benign immune response; however, there are no specific markers to identify patients requiring additional treatment [20].

Overall, there is a favorable visual prognosis for syphilitic posterior segment manifestations with early and appropriate treatment [12, 17]. However, one study by Bollemeijer and colleagues showed that although there was a statistically significant improvement in VA at 1 and 6 months in patients treated with IV antibiotics and adjunct corticosteroids, 13.5% reported continued chronic uveitis with no relation to the duration of treatment delay [12]. Hamann et al. corroborated these results, highlighting that over a third of eyes affected with ocular syphilis had persistent abnormal vision despite antibiotic treatment [21]. Although most cases of ocular syphilis may result in a good visual outcome after treatment, some cases may have lasting visual disturbances, as demonstrated in our study.

## Conclusion

Management of ocular syphilis is challenging despite the efficacy of treatment with penicillin. In our case series, we documented three syphilis cases with persistent retinal manifestations despite following standard of care. These cases demonstrated chronic inflammation, incomplete treatment, and reinfection as contributors to the persistence. Other factors associated with poor visual prognosis include treatment delay, poor presenting VA, and macular edema. In HIV patients, there is an increased risk of recurrence. In cases of persistent ocular findings following treatment, we recommend first checking for treatment completion and a decline in RPR titers, then beginning anti-inflammatory therapy. It is important to note, however, that serological titers may not always be reliable for confirming disease resolution.

## Data Availability

No datasets were generated or analysed during the current study.

## Abbreviations

SOR: Syphilitic Outer Retinopathy
ASPPC: Acute Syphilitic Posterior Placoid Chorioretinopathy
IV: Intravenous
VA: Visual Acuity
OD: Right Eye
OS: Left Eye
OU: Both eyes
FAF: Fundus Autofluorescence
OCT: Optical Coherence Tomography
CME: Cystoid Macular Edema
RPE: Retinal Pigment Epithelium
EZ: Ellipsoid Zone
ELM: External Limiting Membrane
RPR: Rapid Plasma Regain
CF: Counting Fingers
HIV: Human Immunodeficiency Virus
HM: Hand Motion
